# A comparative study of total knee arthroplasty outcome for stiff knee with or without sequential antirheumatic drug treatment

**DOI:** 10.1186/s13018-021-02662-5

**Published:** 2021-08-21

**Authors:** Cheng-Qi Jia, Xiao-Rui Guan, Zhi-Lai Zhao, Ji-Ying Chen, Xiang Li

**Affiliations:** 1 Department of Orthopedics, Chinese PLA General Hospital, Beijing, China; 2 Department of Orthopedics, 541 General Hospital, Shanxi, China; 3 Department of Orthopadics, Liaocheng Veterans Hospital, Shandong, China

**Keywords:** Stiff knee, Rheumatoid arthritis, Ankylosing spondylitis, Total knee arthroplasty, Sequential antirheumatic drug treatment

## Abstract

**Background:**

The aim of this study was to evaluate the influence of antirheumatic drug treatment on knee function of stiff knee patients after total knee arthroplasty (TKA).

**Methods:**

Twenty-seven patients (44 knees) of active RA (rheumatoid arthritis) or AS (ankylosing spondylitis) with stiff knees were included in this study. And they were divided into two groups according to continue antirheumatic drug treatment or not after TKA: the therapeutic group (16 patients, 27 knees) and the controlled group (11 patients, 17 knees). The outcomes were assessed by Knee Society Score (KSS), Visual Analogue Scale (VAS), range of motion (ROM) (at week 6, month 6, year 1, and year 2), “Forgotten Joint” Scale (FJS), with or without crutch, satisfaction, and revision (at year 2). The knee prosthetic loosening was evaluated by the followed X-ray at each following time.

**Results:**

The mean follow-up time was 51 months (34–69 months). The KSS was higher at week 6 after TKA in the therapeutic group (*p* < 0.05); however, the functional scores of KSS at month 6, year 1, and year 2 in the controlled group were more points improved. The therapeutic patients preferred the knee more at month 6, year 1, and year 2. The differences of KSS clinical scores (at month 6, year 1, and year 2), VAS, ROM, Crutch and FJS between the two groups were not statistically significant (*p* > 0.05).

**Conclusion:**

For patients with stiff knees, the sequential antirheumatic drug treatment after TKA had no obvious effect on postoperative KSS, but can improve the satisfaction.

**Level of evidence:**

Therapeutic level II. See Instructions for Authors for a complete description of levels of evidence.

## Introduction

The amount of flexion achieved after total knee arthroplasty (TKA) is determined by the amount of preoperative flexion, especially if the flexion was less than 50° [1]. Therefore, preoperative restriction of knee motion is a challenge for surgeons [2].

Stiffness can be defined as limited range of motion (ROM) of 50° or less that affects a patient’s ability to perform activities of daily living. One study found patients require an average of 83° knee flexion to climb stairs foot over foot. To sit in a chair without using one’s hands requires 93° knee flexion on average, and tying one’s shoes while seated requires 106° flexion on average [1].

There were some studies regarding results of primary TKAs in patients with a preoperative arc of motion of 50° or less. From these literatures, we knew that the surgical approach in these patients could be a challenge because of difficulty in patellar eversion. Technical problems include lack of adequate exposure, the need for extensile surgical approaches, the risk of patellar tendon avulsion, difficulty balancing the flexion–extension gaps, component malpositioning, extensor mechanism management, patellar maltracking, avulsion of the collateral ligaments, and difficulty in wound closure [3]. However, others have reported that postoperative ROM in patients with stiff knees can be the same as ROM in patients with flexible knees, and that there were low complication rates in these replaced knees [4]. Numerous studies have mainly focused on the stiff knee [1]; there may be a tendency to underestimate the outcome for stiff knee with or without sequential drug treatment for rheumatoid and ankylosing spondylitis.

We asked whether the sequential antirheumatic drug after TKA influences knee function, ROM, and satisfaction for stiff knee patients.

## Methods

### Study design

In clinical work, it was found that some patients with RA or AS after TKA had spontaneously stopped rheumatology drug treatment for economic reasons or autonomous activity. We collected 30 patients with stiff knees and started follow-up observation from week 6 after TKA, when the control group stopped all postoperative and antirheumatic drug treatment.

The inclusion criteria were as follows: (1) aged between 18 and 65, (2) all surgical procedures were conducted by the same surgeon, (3) all patients had a preoperative flexion arc of 50° or less in knees, and (4) a minimum follow-up period of 2 years. The exclusion criteria were as follows: (1) a history of septic arthritis of the knee or osteomyelitis, (2) any medical disability that limited the ability to walk and would not be considered suitable for a minimum 2-year follow-up period, (3) disabling diseases involving other joints of the lower extremities and severe deformities (varus angulation, valgus angulation, or flexion contracture of more than 15°), (4) mental diseases, and (5) patients participating in other trials [5].

**Fig. 1:**
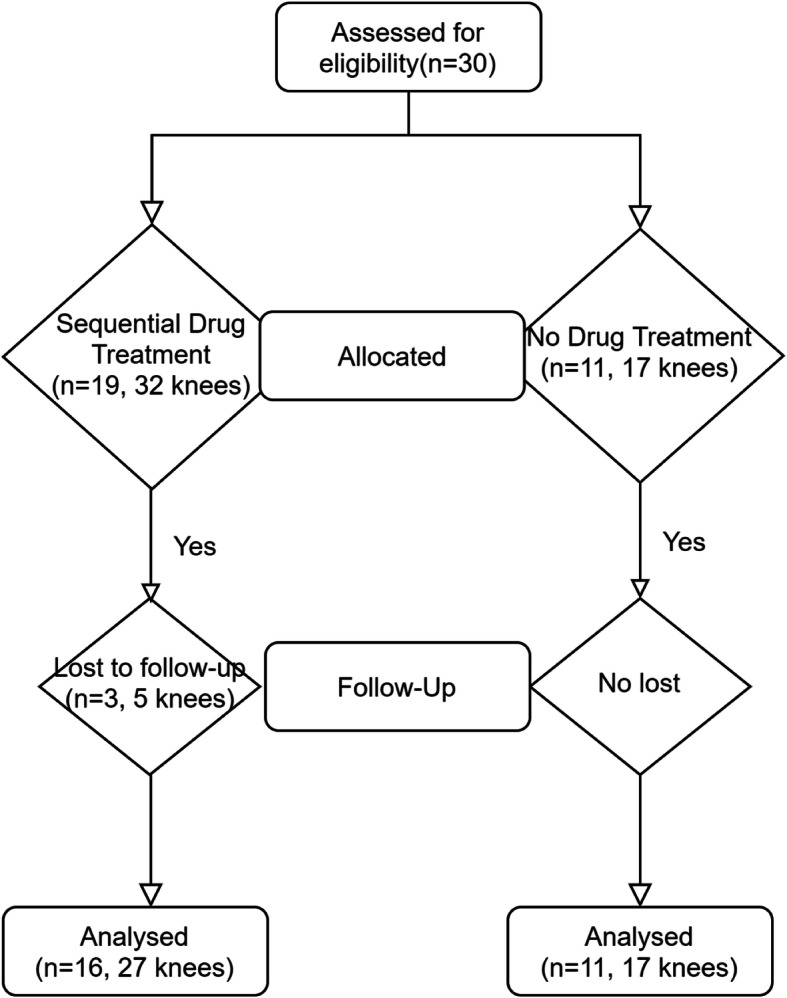
Flow diagram

These TKAs were performed by a senior author from January 2015 to October 2017. The patients were classified into two groups: Group 1 included stiff knees with sequential drug treatment after TKA (3 patients lost to follow-up; 27 knees in 16 patients; bilateral in 11 patients and unilateral in 5 patients), and Group 2 included stiff knees without sequential drug treatment (17 knees in 11 patients; bilateral in 6 patients and unilateral in 5 patients) (Fig. 1).

We evaluated the patients preoperatively and postoperatively at intervals of 6 weeks, 6 months, 1 year, and yearly thereafter until the final follow-up. Preoperative and postoperative clinical evaluations were performed by two independent orthopedic surgeons according to KSS, ROM, VAS, FJS, and Crutch. Data results are cross-checked by the other two independent orthopedic surgeons.

The preoperative data included the patient demographics (Table 1), ROM, KSS, and VAS (Tables 1, 2, 3, and 4). There were 12 women and 15 men. Ten patients had simultaneous bilateral TKAs under one anesthesia. Seven patients had staged bilateral TKAs, and 10 had a unilateral TKA.

**Table 1:** Patient demographic parameters

Parameters	Drug treatment (*N* = 16, 27 knees)	No drug treatment (*N* = 11, 17 knees)	*p value*
Age^**a**^**,** year	39.56 ± 11.74	38.64±9.21	0.828
Gender			
Male, no. (%)	8 (50%)	7(63.63%)	0.696
Female, no.(%)	8 (50%)	4(36.36%)	
Height^**a**^, cm	163.94 ± 12.15	162.82±8.32	0.793
Weight^**a**^**,** kg	62.29 ± 15.61	54.00±8.34	0.121
BMI^**a**^, kg/m^2^	22.97 ± 4.15	20.44±3.25	0.102
Diagnosis			1.000
Rheumatoid arthritis	9	7	
Ankylosing spondylitis	7	4	
Knee			
Left knee	13	10	0.548
Right knee	14	7	
Bilateral TKAs	11	6	
Unilateral TKA	5	5	
Inpatient days^**b**^**,** days	13.5 (6–29)	12 (7–28)	0.568
Follow-up^**b**^**,** months	46.5 (35–69)	58 (34–69)	0.138
Crutch, no. (%)			
With crutch	7 (43.75%)	2 (18.18%)	0.231
Without crutch	9 (56.25%)	9 (81.82%)	
Revision knee, no. (%)	2 (2/44, 2.27%)	0	1.000

*BMI* body mass index

^a^The values are given as the mean and the standard deviation

^b^The values are given as the median, with the range in parentheses

**Table 2:** Comparison of operative data between two groups after TKA

Operative data	Drug treatment (*N* = 16, 27 knees)	No drug treatment (*N* = 11, 17 knees)	*p value*
Surgical time^**a**^**,** min	122.5 (60–240)	127.5 (62.5–285)	0.904
Blood loss^**a**^**,** ml	300 (50–600)	250 (50–600)	0.565
ASA			0.624
II	13	10	
III	3	1	
Prosthesis			0.333
Depuy PFC	11	9	
Depuy PS150	6	1	
Depuy RP	4	5	
Link RK	6	2	
Patella replacement, no. (%)	8 (28.57%)	3 (17.65%)	0.592
Soft tissue and bone procedures			
The fused knee	8	5	
VY Quadricepsplasty	4	0	
Iliotibial band lysis	3	0	
Plus osteotomy (femoral)	12	8	
Plus osteotomy (tibia)	0	2	
Residual flexion deformity, no. (°)	1 (15°)	2 (15°)	

^a^The values are given as the median, with the range in parentheses

**Table 3:** Comparison of KSS score between two groups after TKA

		Clinical score					F1unctional score			
	Preoperative	Postoperative at week 6	Postoperative at month 6	Postoperative at year 1	Postoperative at year 2	Preoperative	Postoperative at week 6	Postoperative at month 6	Postoperative at year 1	Postoperative at year 2
Group										
Drug treatment^**a**^	9 (0–69)	0 (0–5)	83 (56–99)	83 (56-99)	85 (37–99)	73 (32–84)	5 (0–70)	55 (5–100)	60 (20–100)	60 (20–100)
No drug treatment^**a**^	0 (0–52)	5 (0–5)	89 (53–99)	89 (52–99)	89 (52–99)	54 (32–78)	0 (0–55)	100 (0–100)	100 (0–100)	100 (0–100)
Statistic	*p* = 0.084	*p* = 0.026*	*p* = 0.068	*p* = 0.068	*p* = 0.184	*p* = 0.10	*p* = 0.026*	*p* = 0.002*	*p* = 0.003*	*p* = 0.003*

^a^The values are given as the median, with the range in parentheses. * p < 0.05.

**Table 4:** Comparison of ROM, VAS, and FJS between two groups after TKA

		ROM					VAS				FJS
	Preoperative	Postoperative at week 6	Postoperative at month 6	Postoperative at year 1	Postoperative at year 2	Preoperative	Postoperative at week 6	Postoperative at month 6	Postoperative at year 1	Postoperative at year 2	Postoperative at year 2
Group											
Drug treatment^**a**^	10 (0–50)	90 (30–95)	85 (30–110)	80 (30–110)	80 (30–110)	6 (0–9)	3 (0–9)	0 (0–5)	0 (0–5)	0 (0–5)	50 (25–100)
No drug treatment^**a**^	25 (0–50)	90 (60–90)	90 (60–90)	90 (60–90)	90 (70–90)	6 (3–9)	3 (0–6)	0 (0–4)	0 (0–4)	0 (0–4)	75 (25–75)
Statistic	*p* = 0.435	*p* = 0.658	*p* = 0.627	*p* = 0.332	*p* = 0.284	*p* = 0.335	*p* = 0.188	*p* = 0.065	*p* = 0.158	*p* = 0.351	*p* = 0.335

*ROM* range of motion*, VAS* visual analogue scale*, FJS “*Forgotten Joint” Scale

^a^The values are given as the median, with the range in parentheses

The operative data included operation time, intraoperative blood loss, prosthesis type, patella replacement, and soft tissue and bone procedures (Table 2).

Postoperatively, we not only studied the KSS, ROM, VAS, with or without crutch, and radiographic data of patients, but also the revision rate, self-satisfaction, and “Forgotten Joint” Scale (FJS) (Tables 1, 2, 3, 4 and 5). Patients’ satisfaction was classified as Very good if they have no other uncomfortable feelings, Good if they have few special feelings, General if they could accept some uncomfortable feelings, and Not good if they could not accept the uncomfortable feelings.

**Table 5:** Comparison of FJS and the patients’ satisfaction between two groups after TKA, no. (%)

		Patients’ satisfaction			
	Grade	Postoperative at week 6	Postoperative at month 6	Postoperative at year 1	Postoperative at year 2
Group					
Drug treatment	Very good^**a**^	1 (1/27)	1 (1/27)	1 (1/27)	1 (1/27)
	Good^**a**^	15 (15/27)	11 (11/27)	11 (11/27)	11 (11/27)
	General^**a**^	11 (11/27)	13 (13/27)	13 (13/27)	13 (13/27)
	Not good^**a**^	0 (0/27)	2 (2/27)	2 (2/27)	2 (2/27)
No drug treatment	Very good^**a**^	0 (0/17)	0 (0/17)	0 (0/17)	0 (0/17)
	Good^**a**^	8 (8/17)	2 (2/17)	2 (2/17)	2 (2/17)
	General^**a**^	6 (6/17)	4(4/17)	4 (4/17)	4 (4/17)
	Not good^**a**^	3 (3/17)	11 (11/17)	11 (11/17)	11 (11/17)
Statistic		*p* = 0.00*	*p* = 0.035*	*p* = 0.00*	*p* = 0.00*

^a^These indexes of the patients’ satisfaction were classified as Very good if they have no other uncomfortable feelings, Good if they have few special feelings, General if they could accept some uncomfortable feelings, and Not Good if they could not accept the uncomfortable feelings. *p < 0.05.

Radiographic evaluations of alignment were performed on preoperative and follow-up postoperative radiographs, which included anteroposterior (AP) standing, lateral and skyline patellar views, and full length of lower limbs. The data was evaluated by another independent orthopedic surgeon who did not know the patient’s sequential drug treatment. Data regarding the intraoperative and immediate postoperative complications were retrieved from the operating notes. Revision for any reason was documented.

### Operation procedures

All patients received the total knee arthroplasty (Depuy PFC, Depuy RP, Depuy PS150, Link RK). The surgeries were performed by one senior physician under tourniquet control using a medial parapatellar approach. The posterior cruciate ligament was sacrificed because of severe deformity. We used an extramedullary alignment jig for the tibia and an intramedullary alignment jig for the femur. The patella was replaced in 11 knees (8 knees in group 1; 3 knees in group 2). We performed a patelloplasty, which included soft tissue release from the lateral patella, division of the patellofemoral ligament, and patellar rim cautery to provide partial denervation and osteophyte removal. We removed 2–4 mm of the articular surface of the patella in TKA [2].

We performed an extensile exposure (rectus snip) in the knees to enable eversion of the patella and knee flexion. Soft tissue procedures included lateral retinacular release and lateral gutter debridement [6]. Extensive medial capsular sleeve dissection, including stripping the superficial medial collateral ligament, was performed in the knees to correct the preoperative varus deformity. The iliotibial band was released from Gerdy’s tubercle in 3 knees with a preoperative valgus deformity. In 4 knees, we performed a quadriceps VY-plasty early during exposure to facilitate patellar eversion. The VY-plasty was repaired with the knee in 45° flexion to prevent extension lag (Table 2). A subperiosteal femoral peel was needed in all knees. All knees required extensive posterior capsulotomy and subperiosteal elevation of the gastrocnemius from the posterior femur. Twenty-three knees required an additional distal femoral cut (20 knees) or proximal tibia cut (2 knees) to correct the severe preoperative flexion deformity. Posterior stabilized components were used in 36 knees (20 Depuy PFC, 9 Depuy RP, 7 Depuy PS150), and a constrained condylar prosthesis (Link RK) was used in 8 knees, with an asymmetric laxity greater than 1 cm for the capsular and ligamentous insufficiency (Table 2). All patients had at least a 90° arc of flexion after wound closure. The patella was fused to the anterior femur in 13 patients (Table 2) and was osteotomized and reflected laterally after arthrotomy (Table 2). The tibiofemoral joint line was visible in all knees, even in patients with bony ankylosis. We performed the osteotomy along the joint line using a curved osteotome to separate the tibia and femur. We used precaution to preserve as much bone as possible and to preserve the medial and lateral soft tissue sleeve.

Patients received antibiotic prophylaxis with intravenous ceftriaxone sodium (2 g, 30 min before tourniquet followed by 2 g for the next day). If the operation time exceeds 3 h or the blood loss is greater than 1500 ml, a second dose can be given during the operation, but no antithrombotic prophylaxis (according to the special blood state of RA and AS patients). The postoperative regimen included intravenous and oral analgesia (oral until 6 weeks after TKA), knee extension training immediately, gravity-assisted regaining of flexion, and walking with support from the second postoperative day. Progressive resistance exercises were started at day 2 postoperatively to strengthen the quadriceps and continued for 1 year postoperatively. All patients used walkers in 6 weeks postoperatively. The main drugs used in the sequential drug treatment group after TKA are methotrexate, sulfasalazine, tripterygium wilfordii, steroid, leflunomide, nonsteroidal anti-inflammation drugs, etanercept, and root of herbaceous peony (Table 6) [6].

**Table 6:** The sequential drug treatment of group 1 after TKA

Serial number	Number of knees	Diagnosis	Drug 1	Drug 2	Drug 3	Drug 4
1	2	AS	NSAIDS	Sulfasalazine	Methotrexate	Root of herbaceous peony
2	2	AS	NSAIDS			
3	2	RA	Tripterygium wilfordii	Prednisone		
4	1	RA	Traditional Chinese Medicine			
5	2	RA	NSAIDS	Leflunomide		
6	1	RA	NSAIDS	Leflunomide	Iguratimod	
7	1	RA	NSAIDS	Leflunomide	Iguratimod	
8	2	AS	NSAIDS			
9	2	AS	NSAIDS	Leflunomide	Iguratimod	
10	2	RA	NSAIDS	Huangteng		
11	1	RA	NSAIDS	Iguratimod	Sulfasalazine	
12	2	RA	NSAIDS	Sulfasalazine	Leflunomide	
13	2	AS	NSAIDS	Leflunomide		
14	2	AS	Etanercept			
15	1	AS	NSAIDS			
16	2	RA	NSAIDS	Methotrexate	Sulfasalazine	Tripterygium wilfordii
16	27					

*RA* rheumatoid arthritis*, AS* ankylosing spondylitis*, NSAIDS* nonsteroidal anti-inflammatory drugs

### Statistical analysis

SPSS 24.0 (SPSS Inc) was used for statistical analysis by an independent orthopedic surgeon. Clinical data were analyzed using means ± standard deviation or the median with the range in parentheses. The level of statistical significance was defined as *p* < 0.05. Independent sample *t* tests were performed to determine the difference in age and body mass index (BMI). We used the Mann–Whitney test of nonparametric test for continuous variables when the data did not conform to the normal distribution, like the duration of hospital stay, operation time, blood loss, KSS, VAS, ROM, and FJS. Chi-squared test or Fisher's exact probability test were for categorical variables, like satisfaction and Crutch.

## Results

We observed no differences in revision rates between the two groups (Table 1). In group 1, there was 1 patient (2 knees) whose knee prosthesis was successively revised due to aseptic loosening at year 2 after the first TKA (Table 1). There were 3 cases of residual 15° flexion deformity after TKA (1 in group I, 2 in group 2) (Table 2).

**Fig. 2:**
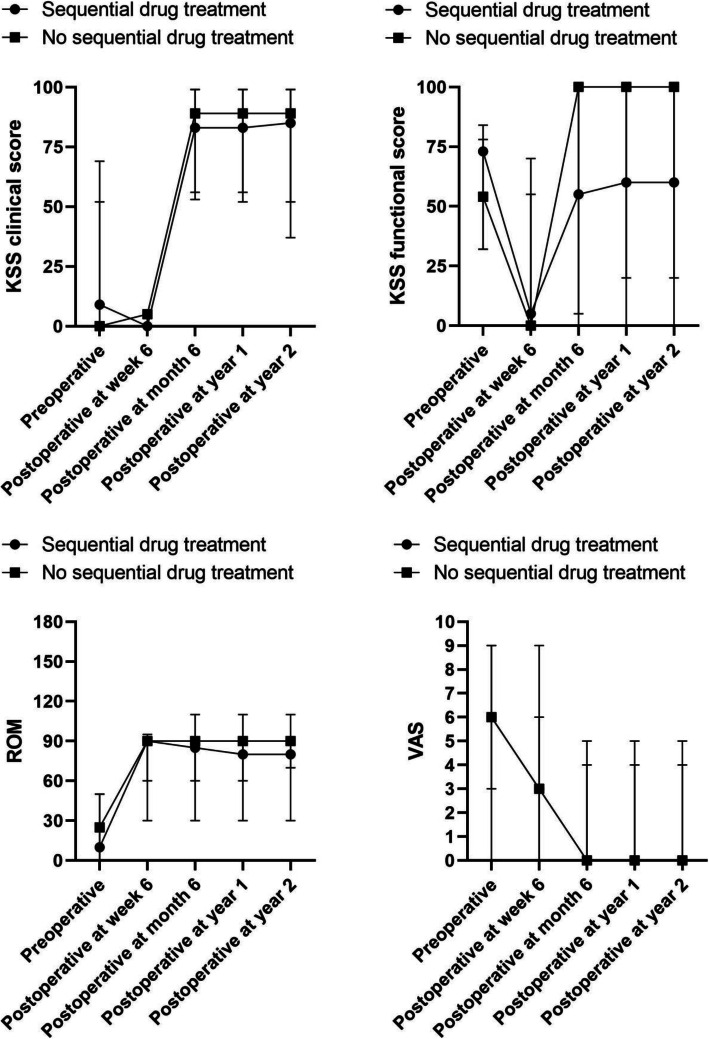
Changes in KSS, ROM, and VAS at dfferent follow-up time

The KSS in group 1 was higher than that in group 2 postoperatively at week 6 (*p* < 0.05) (Table 3). However, the postoperative functional scores of KSS in patients with sequential drug treatment (group 1) were lower (*p* < 0.05) than that in patients without sequential drug treatment (group 2) at month 6, year 1, and year 2 (Fig. 2). The clinical scores of KSS (at month 6, year 1, and year 2) between the two groups were not statistically significant (*p* > 0.05) (Fig. 2).

The median preoperative flexion deformity was 10° (range, 0–50°) in group 1 and 25° (range, 0–50°) in group 2, without statistical significance (*p* = 0.435) (Table 4). Postoperatively, the arc of motion at different following time in two groups were not statistically significant (*p* > 0.05) (Fig. 2).

The median preoperative VAS was 6 (range, 0–9) in group 1 and 6 (range, 3–9) in group 2, without statistical significance (*p* = 0.335) (Table 4). Postoperatively, VAS at different following time in two groups were not statistically significant (*p* > 0.05) (Fig. 2).

FJS scores at year 2 were 50 (range, 25–100) in group 1 and 75 (range, 25–75) in group 2 (Table 4). The difference between the two groups was not statistically significant (*p* = 0.335).

And the differences of Crutch between the two groups were not statistically significant (*p* = 0.231) (Table 1). After TKA, there were 7 patients in the group of sequential antirheumatic drugs (group 1) who still used crutches at the 2-year follow-up and 2 patients in the other group (group 2) (Table 1).

However, patients in group 1 were more satisfied than those in group 2 at month 6, year 1, and year 2 after TKA (*p* < 0.05) (Table 5). The imaging results are normal.

## Discussion

Early treatment of RA and AS can effectively prevent joint destruction and improve the quality of life by controlling symptoms and inflammation. The main antirheumatic drugs used are (1) Nonsteroidal anti-inflammation drugs; (2) glucocorticoids, local injection to treat refractory peripheral arthritis; (3) rheumatology medication, the drugs recommended by the American Academy of Rheumatology classification standards mainly include sulfasalazine and methotrexate and leflunomide, etc.; (4) biological agents, TNF antagonists are the first choice, which can improve the pain and function of the axial joints; (5) thalidomide, for special patients (when other related drugs have no effect, these drugs can significantly improve clinical symptoms) [7].

Due to severe pain, restricted mobility, and deformities in patients with advanced RA and AS, medical treatment can no longer meet the needs of patients; therefore, TKA is the best choice [1] as patients with RA and AS have relatively young age, active lifestyles, and higher requirements for postoperative results, in addition to reasonable rehabilitation exercises, whether the continued use of drug therapy after TKA can improve knee function. To verify this idea, we conducted follow-up observations on these two types of patients.

Our series (44 stiff knees: 27 with sequential drug treatment versus 17 without sequential drug treatment of knees) was the first series of TKAs in stiff patients with limited ROM of 50° or less to verify this question. Our data observed that stiff knee patients with sequential antirheumatic drug treatment after TKA had a better clinical and functional outcome at week 6 after TKA than patients without antirheumatic drug treatment, but had lower scores in the functional part of KSS at month 6, year 1, and year 2. The clinical scores of KSS at other follow-up time were not statistically significant (*p* > 0.05).

We considered that under taking analgesia in both groups in week 6 after TKA, patients sequentially taking antirheumatic drugs at the same time had a greater degree of pain relief and local inflammation control, thereby better improving their postoperative clinical and functional scores of KSS at week 6 after TKA. Then the two groups of patients stopped using conventional analgesics simultaneously; patients in group 1 continued antirheumatic drug treatment, and patients in group 2 stopped taking antirheumatic drugs due to economic problems or active exercise awareness. According to follow-up, we believed that under economic pressure or active exercise awareness, their (in group 2) activity level would increase in order to recover quickly, which made them have higher KSS functional scores at month 6, year 1, and year 2 after TKA. This situation was speculated to a certain extent that the KSS after TKA is related to the amount of activity.

There was not much published information regarding results of TKAs in knees with a preoperative arc of motion less than 50°. Aglietti and Buzzi (20 stiff knees, six ankylosed knees) reported ankylosed knees achieved less motion than stiff knees [3]. Montgomery et al. reported no difference in the results after TKA when comparing ankylosed knees with knees of relatively normal motion [2]. Dr. Ashok Rajgopal (96 patients, 115 knees) reported the long-term functional outcome scores of stiff knees in extension and in flexion are similar [8]. However, no studies included stiff knee patients to accurately compare sequential antirheumatic drug treatment after TKA with no sequential antirheumatic drug treatment after TKA to identify the knee functional improvement.

Postoperative arc of flexion ranging from 64 to 103° had been reported in stiff and ankylosed knees in series ranging from 3 to 84 knees [9]. Our results were comparable to those of previous studies.

In our study, the median postoperative arc of motion at different following time in two groups were not statistically significant (*p* > 0.05) (Table 4), but the median and mean ROM of patients who did not use antirheumatic drugs (group 2) were both higher than those who used antirheumatic drugs (group 1) at month 6, year1, and year 2. This may indicate that patients who did not take antirheumatic drugs sequentially had greater mobility of their knee joints because of active rehabilitation activities.

Postoperatively, VAS at different following time in two groups were not statistically significant (*p* > 0.05) (Table 4). But the mean VAS in group 1 were higher than those in group 2 at month 6, year 1, and year 2. This may indicate that patients with sequential use of antirheumatic drugs were more sensitive to pain, which limited their postoperative activity.

FJS scores at year 2 were 50 (range, 25–100) in group 1 and 75 (range, 25–75) in group 2 (Table 4) (*p* = 0.335). This may indicate that patients with sequential antirheumatic drug treatment after TKA did not make sense to forget joint replacement.

In terms of higher satisfaction after TKA in patients with sequential drug treatment, we considered that patients who took antirheumatic drugs continually (at week 6, month 6, year 1, and year 2) after TKA would help relieve their general discomfort and control local inflammation (Table 5).

In addition, small bone sizes, severe osteopenia in a high proportion of patients with RA and AS, and severe soft tissue contractures made the operation technically demanding [10]. During exposure, stiff knees may need a rectus snip for exposure. Knees with osseous ankylosis in extension almost always need VY quadricepsplasty before the patella can be everted to minimize the risk of patellar tendon avulsion [11]. Aglietti and Buzzi recommended early quadricepsplasty to aid in exposure and patellar eversion without compromising the integrity of the patellar tendon [3]. We performed early quadricepsplasty in 4 knees with osseous ankylosis in extension. Twenty knees underwent plus osteotomy on the femoral side, and two knees underwent plus osteotomy on the tibial side. One patient underwent quadriceps garter treatment. Three cases of knee joints underwent iliotibial band lysis. All patients underwent subperiosteal dissection (Table 2).

A constrained total knee prosthesis has been recommended for converting a fused knee to a TKA to substitute for deficient or absent collateral ligaments [12]. In our experience, posterior-stabilized prostheses were also performed successfully in patients with stiff knees.

McAuley et al. evaluated 27 TKAs in patients with a preoperative range of flexion less than 50° [4]. They reported an overall complication rate of 41% with a revision rate of 18.5%. Similar high complication and revision rates were reported by Naranja et al. in patients who had TKAs for ankylosed knees [13]. The overall revision rate in our series (2.27%) was lower than rates in previous studies.

Our study has several limitations. The primary limitation is that the study lacked adequate power to compare the results of TKAs in patients with knees ankylosed in extension with the results in patients with knees ankylosed in flexion. The other limitation is that several prostheses were used during the study, and the comparison of results based on the implant used also would be prone to inadequate power. To sit in a chair without using one’s hands requires 93° knee flexion on average, and tying one’s shoes while seated requires 106° flexion on average; more cases are needed for subgroup analysis from the two angles [8].

Our results were inferior to results of a standard primary TKA and had a lower KSS and FJS [14]. The surgery is technically demanding and should be performed only by a surgeon with considerable experience. Patients need to be counseled preoperatively regarding the possibility of a suboptimal outcome compared with that of a standard TKA performed in a mobile knee; the need for prolonged physiotherapy, more activity and higher tolerance; and the high complication rate.

In conclusion, for patients with stiff knees, the sequential antirheumatic drug treatment after TKA had no obvious effect on postoperative KSS, but can improve the satisfaction. And according to the result of a higher postoperative functional values of KSS in patients without sequential drug treatment, we considered more postoperative activity or better active awareness can improve postoperative function. We recommend patients with RA or AS undergo more activity in time after TKA.

## Conclusion

For patients with stiff knees, the sequential antirheumatic drug treatment after TKA had no obvious effect on postoperative KSS, but can improve the satisfaction.

## Data Availability

We do not wish to share our data, because some of the patient’s data regard individual privacy, and according to the policy of our hospital, the data could not be shared with others without permission.

## Abbreviations

RA: Rheumatoid arthritis
AS: Ankylosing spondylitis
TKA: Total knee arthroplasty
NSAIDS: Nonsteroidal anti-inflammatory drugs
KSS: Knee society score
BMI: Body mass index
ROM: Range of motion
VAS: Visual analogue scale
FJS: “Forgotten Joint” Scale
AP standing: Anteroposterior standing
